# 

**Published:** 1907-01-15

**Authors:** 


CONTENTS

Event and Comment - - - - - - - - - - 1

Re-Organization of the Illinois State Dental Society and the Application of the Plan in Michigan, *By Arthur D. Black, B.S., M.D., D.D.S.* 5

The Detrimental Physical Properties of Expansion and Compression of Plaster of Paris, - *By J. H. Prothero, D.D.S.* 22

With Our Editors - - - - - - - - - - 37

Indents - - - - - - - - - - - 45

Announcements - - - - - - - - - - 52

